# Placebo Responses and Their Clinical Implications in Fibromyalgia: A Meta-Analysis Using SSRI and SNRI Trials

**DOI:** 10.3389/fpain.2021.750523

**Published:** 2021-12-07

**Authors:** Helen Koechlin, Anna Kharko, Tamara Probst, Julia Pradela, Stefan Buechi, Cosima Locher

**Affiliations:** ^1^Division of Clinical Psychology and Psychotherapy, Faculty of Psychology, University of Basel, Basel, Switzerland; ^2^Department of Anesthesiology, Critical Care and Pain Medicine, Boston Children's Hospital and Harvard Medical School, Boston, MA, United States; ^3^Faculty of Health, University of Plymouth, Plymouth, United Kingdom; ^4^Clinic for Psychotherapy and Psychosomatics “Hohenegg”, Meilen, Switzerland; ^5^Department of Consultation-Liaison Psychiatry and Psychosomatic Medicine, University Hospital Zurich, Zurich, Switzerland

**Keywords:** placebo, fibromyalgia, antidepressants, SSRIs, SNRIs, meta-analysis

## Abstract

**Background:** Fibromyalgia (FM) is a chronic primary pain condition, associated with widespread musculoskeletal pain, disturbed sleep, fatigue, cognitive dysfunction, and a range of comorbid conditions such as irritable bowel syndrome, and depression. Despite its high prevalence of 2% in the general population, FM continues to pose scientific and clinical challenges in definition, etiology, and day-to-day management. In terms of treatment, FM can be treated with selective serotonin reuptake inhibitors (SSRIs), serotonin-norepinephrine reuptake inhibitors (SNRIs).

**Objective:** Patients with FM and other chronic primary pain syndromes are known to experience substantial and clinically relevant placebo effects. An update of the placebo responses for various outcomes in the FM population and especially a discussion about clinical implications is therefore needed.

**Methods:** We used data from a large data pool that includes randomized controlled trials (RCTs) examining within-placebo mean change scores of baseline vs. follow-up assessments in FM trials of SSRIs and SNRIs. The primary outcomes were pain, functional disability, and depression and using different scales. We assessed heterogeneity of included trials.

**Results:** A total of 29 RCTs with *N* = 8,453 patients suffering from FM were included in our analysis. Within-placebo mean change scores of baseline vs. follow-up assessments were large for pain (mean change = 2.31, 95% CI: 0.42–4.21, *p* = 0.017), functional disability (mean change = 3.31, 95% CI: 2.37–4.26, *p* < 0.000), and depression (mean change = 1.55, 95% CI: 0.92–2.18, *p* < 0.000). Heterogeneity was found to be large for all outcomes.

**Impact:** Our results provide preliminary evidence that placebo responses, which also consist of non-specific effects, might play a role in the treatment of FM. Furthermore, we highlight limitations of our analyses and make suggestions for future studies.

## Introduction

Fibromyalgia (FM) is a Chronic Primary Pain condition classified under MG30.01 Chronic Widespread Pain in the ICD-11. The cardinal markers of FM include non-specific musculoskeletal pain, fatigue, chronically disturbed sleep, and mild cognitive dysfunction (1). FM is common, with estimated prevalence in the general population to be between 2 and 10% (2), and a majority of patients being female (3). Significant challenges, however, in the diagnosis and long-term management of the syndrome persist. On paper, since 1990 reaching a diagnosis has relied on the American College of Rheumatology (ACR) criteria, which are regularly updated to better the quantification of the central FM symptoms and comorbidities (4–6). In clinical practice, both poor knowledge of (7) and poor adherence (8) to the ACR criteria has been observed. Instead, in line with newer recommendations, differentiation (9) from symptomatically similar conditions such as such as somatic or rheumatic diseases and a comprehensive review of patient history drive diagnosis (10).

It remains undetermined what causes FM but separate mechanisms have been suggested for the individual symptoms. Chronic pain, for example, has been linked to central sensitization, a physiological process, in which nociceptive input is abnormally amplified in dorsal horn neurons (11). This leads to both allodynia, perception of otherwise innocuous stimuli as painful, and hyperalgesia, the heightened sensitivity to painful stimuli. A more comprehensive explanation of FM is offered by the biopsychosocial framework, which acknowledges the interactive contribution of biological, psychological and social factors to the syndrome (12). Importantly, it proposes that concurrent management of affective distress such as depression is an integral part of managing FM (13). Still, long-term treatment strategies are effectively reduced to management of individual symptoms, guided by patient treatment preferences (14) and thus lack global standardization (15). Central healthcare goal is pain management (16) and it is commonly addressed through pharmacological interventions.

There are several options for pain management through pharmacotherapy in FM. The most common include non-steroidal anti-inflammatory drugs (NSAIDs), selective serotonin reuptake inhibitors (SSRIs), serotonin-norepinephrine reuptake inhibitors (SNRIs), tricyclic antidepressants (TCAs) and gabapentinoids (17). None of these, however, have shown universally beneficial effects in FM patients. NSAIDs, for example, have not been found to be significantly better than the placebos (18). Research on SSRIs and SNRIs, which were deemed promising as they target the typical for FM low serotonin levels, has shown mixed results, with some finding strong evidence for their analgesic efficacy compared to placebo (19), but others failing to find the same (20). Other antidepressants such as tricyclic antidepressants follow a similar pattern with most promising benefits being in terms of sleep quality (21). Opioids, which are not indicated by clinical guidelines but remain common in clinical practice, have been repeatedly rejected in research as a long-term pain management solution due to their lesser effectiveness compared to other medication, but their high incidence of misuse (22). The mixed success rate raises the question if non-specific factors, reflected by the placebo response, have an impact on symptom improvement.

The placebo response is well-established effect across various pharmacological interventions (23). The placebo response is defined as the improvement of patients randomly assigned to the placebo group (24), thus is determined not only by the placebo effect, but also by the natural course of the disease (e.g., spontaneous remission) and statistical artifacts (e.g., regression to the mean) (25). Patients with Chronic Primary Pain (CPP) diagnoses (23), which includes FM, and affective disorders (particularly depression) (26, 27) have been found to be particularly susceptible to placebo (23). Patients with FM have also shown clinically relevant and statistically significant placebo effects (28). However, clinical implications of these findings in the field of FM have only rarely been discussed. A comprehensive assessment of the impact on both pain and concurrent affective distress is needed to reflect the interaction of biopsychological manifestations of FM and the new diagnostic criteria for CPP as stated in the International Classification of Diseases, 11th Edition (ICD-11) (29, 30). Therefore, an updated meta-analysis that takes clinical considerations into account is needed. The main aim of this meta-analysis was to analyze the placebo response in pain, functional disability, and depression in trials examining SSRIs and SNRIs in patients living with FM.

## Methods

### Search Strategy and Study Selection

A systematic literature search of RCTs was undertaken in the following electronic databases: MEDLINE, Embase, PsycINFO, Cochrane Central, and Web of Science, without applying restrictions to language or date of publication. A first search was conducted until April 5, 2018 and was updated in October 2019. The search revealed a total of 72215 records. After removing 9,800 duplicates, 62,415 records remained. Note that the search strategy also included all other categories of CPP (i.e., chronic primary musculoskeletal pain, chronic widespread pain, complex regional pain syndrome, chronic primary headache and orofacial pain, and chronic primary visceral pain), as this analysis is part of a larger project (31). See Appendix 1 for the search strategy for the larger project. Within this larger pool of included RCTs, we went through all full-texts and specifically tagged FM papers, which were then included in the presented analysis. We included RCTs that compare an SSRI and/or an SNRI to a placebo control group or another SSRI and/or SNRI in the treatment of FM. Parallel and crossover trials were included. Protocols and conference papers, randomized single control studies, prophylactic interventions, as well as case-control studies, *post-hoc* analyses or secondary analyses, and results reported solely on clinical trials were excluded. RCTs had to be either in English or German. Patients of both sexes from the age of 18 up, with a primary diagnosis of FM diagnosed by the American College of Rheumatology (ACR) 1990, 2010, or 2016 were included.

### Data Assessment

The following information was extracted from all included studies: study characteristics (lead author, publication year, sponsor, country of study conductance, setting, number of clinical sites), participant characteristics (such as diagnostic criteria, duration of diagnosis, age, sex, duration of symptoms, age of onset, comorbidities), study design (type of study such as parallel or crossover design, special population (if 80% or more of the sample share a particular characteristic), special inclusion criteria, special exclusion criteria, emergency medicine, co-intervention), intervention details (such as a description of the intervention by the authors, provider, treatment duration, dose intended, dose delivered, number of randomized people in the treatment arm, timeframe for post [measured at the time point closest to the end of treatment], timeframe for follow-up 1 [at least 3 months/12 weeks but less or equal to 6 months/24 weeks after randomization], timeframe for follow-up 2 [more than 6 months/25 weeks but less or equal to 12 months/52 weeks after randomization]). If several assessments were reported, we chose the one with the longest timeframe since randomization (i.e., FU2 > FU1 > post). For the continuous outcomes, sample sizes (*N*), means (*M*), standard deviations (SD), CIs, and changes from baseline were noted for each extracted treatment arm of the respective study. If the study reported different doses of either an SSRI or an SNRI, Ms, SDs, and changes were averaged, and *N* was merged. If *N* was reported as a total of all treatment arms, it was divided through the number of treatment arms. Additionally, intention to treat was prioritized over the completer analysis.

As recommended by the Cochrane Handbook for systematic reviews, we always tried to calculate Ms and SDs before imputing them, as imputation methods are based on making assumptions about the trial (32). If a study did not report the mean values numerically, data was extracted from figures using the software DigitizeIt version 2.5 (33). If SDs were not provided, they were calculated from standard errors (SE), *N*, Ms, and/or *p*-values. If SDs could not be calculated, the mean of SDs from studies using the same outcome measure was imputed (34).

### Primary Outcomes

Global pain intensity and the global measurement of pain were our primary outcomes. We extracted both outcomes where both were reported. Additional primary outcomes were a generic measure of functional disability and depression. For all outcomes, we used a pre-defined hierarchy of validated and standardized measurements. For global pain intensity, the hierarchy was as follows: Visual Analog Scale (VAS) > Fibromyalgia Impact Questionnaire (FIQ) (35) > Numeric Rating Scale (NRS) (36); for the global measurement of pain: Brief Pain Inventory (BPI) (37) > Short-Form McGill Pain Questionnaire (SF-MPQ) (38). For emotional distress, we applied the following hierarchy: Beck Depression Inventory (BDI) (39) > FIQ depression subscale (35) > Patient Health Questionnaire 8 (PHQ-8) (40) > Hamilton Depression Rating Scale (HMD) (41) > Montgomery Åsberg Depression Rating Scale (MADRS) (42). For the generic measures of functional disability, studies applied the BPI (37), the Health Assessment Questionnaire (HAQ) (43), or the FIQ (total score or subscale). If different primary outcomes were given in an individual study, the measurement highest in our hierarchy was extracted. The choice of our primary outcomes is in line with recommendations for clinical trials studying chronic pain (IMMPACT initiative) (44). Furthermore, we decided to focus on self-reported measures. Finally, we intend to prioritize global scores over syndrome-specific scores since the definition of CPMP includes various syndromes (45).

### Statistical Analyses

The placebo responses was assessed as the mean change scores of baseline vs. follow-up assessments. A bar chart was created in order to visualize the mean change scores for the placebo group. Analyses were applied within a frequentist framework. We chose to use random-effects models rather than fixed-effects models because the studies that we included were assumed to be heterogenous and the number of included studies was relatively small. Heterogeneity was assessed by calculating the *Q* statistic (46), the τ^2^ (47), and the *I*^2^ (48). An *I*^2^ value of 0% indicates no heterogeneity, a value of 25% is classified as low, 50% as moderate and 75% as high (48).

## Results

### Study Selection

**Figure 1:**
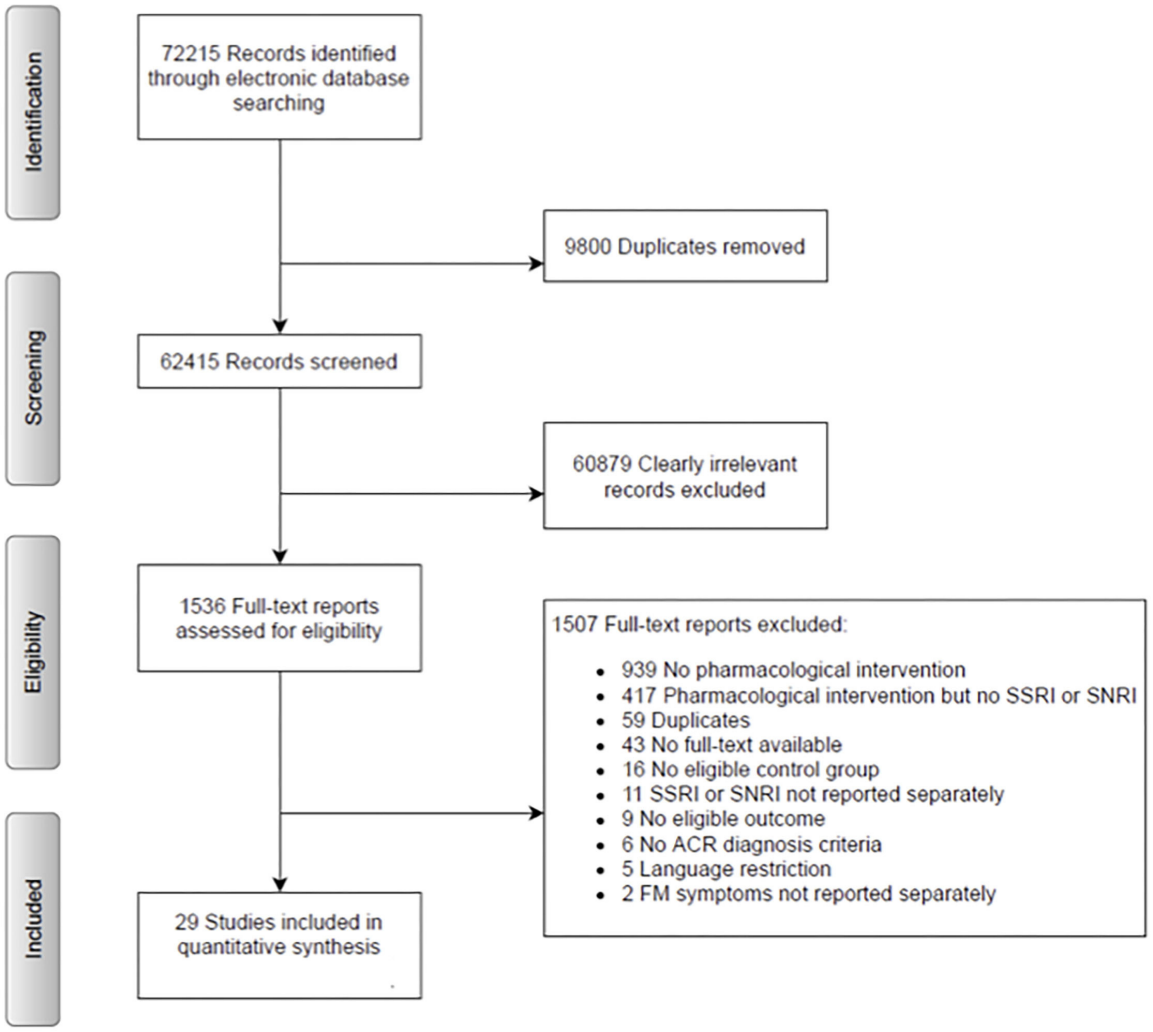
Flow chart.

There were a total of 72,215 identified records for the large project. After removing 9,800 duplicates, 62,415 records were taken into consideration for potential inclusion. For this analysis, 1,536 full texts were screened (see Figure 1). Abstracts and full texts were screened by two independent researchers, consensus was reached in consultation with the first and last author (HK and CL). Finally, 29 RCTs were included in this analysis.

### Study Characteristics

A total of *N* = 8,453 patients were included in the analysis. RCTs were conducted between 1994 and 2018 and compared seven SSRIs and SNRIs with placebo. No study compared two or more pharmacological interventions. Mean sample size was *N* = 146 (*SD* = 184.90). In total, 5,126 (*M* sample size = 176.76, *SD* = 220.42) participants were randomly assigned to pharmacological treatments and 3,327 (*M* sample size = 114.73, *SD* = 137.92) were randomly assigned to placebo. Weighted mean age was 49.15 years. In those studies that reported sex, 94.40% of patients were female. Seventeen of 29 trials (58.62%) recruited patients from the USA, eight from Europe (27.59%), three recruited patients cross continental (10.34%), and one from Asia (3.45%). On average 21.91% of patients suffered from Major Depressive Disorder (MDD). Mean treatment duration was 12.5 weeks (range 4–28 weeks). More detailed information and individual characteristics of the included studies can be found in Table 1. Table 2 shows individual measurements including mean, mean change, and standard deviation for all outcomes across studies.

**Table 1:** Characteristics of included studies.

**Study ID**	**Intervention**	***N* randomized**	**% Female**	**Age**	**Co-intervention**	**Treatment duration (weeks)**	**Timeframe post assessment (weeks)**
Ahmed 2016	Milnacipran Placebo	9 9	89.5% (overall)	49.2 (overall)	Yes	4	4
Allen 2017	Desvenlafaxine Placebo	566 130	93.78% 97.7%	48.6 50.46	NR	15	15
Anderberg 2000	Citalopram Placebo	21 19	100% 100%	48.6 (overall)	Yes	16	16
Arnold 2002	Fluoxetine Placebo	30 30	100% 100%	46 46	Yes	12	12
Arnold 2004	Duloxetine Placebo	104 103	88.5% 89.3%	49.9 48.3	Yes	12	12
Arnold 2005	Duloxetine Placebo	234 120	100% 100%	49.6 (overall)	Yes	12	12
Arnold 2010	Duloxetine Placebo	263 267	92.8% 93.6%	50.7 49.6	Yes	12	12
Arnold 2010	Milnacipran Placebo	516 509	96.9% 93.7%	49.1 48.7	Yes	12	12
Arnold 2012	Duloxetine Placebo	155 153	94.2 96.1	50.9 50.7	Yes	12	12
Branco 2010	Milnacipran Placebo	435 449	95.1% 93.5%	48.3 49.2	Yes	16	16
Chappell 2008	Duloxetine Placebo	162 168	91.98% 94.64%	50.75 50.23	NR	27	27
Clauw 2008	Milnacipran Placebo	802 405	97% 94.8%	49.95 50.7	NR	15	15
Clauw 2013	Milnacipran Placebo	100 51	96% 96%	54.5 54	No	12	12
Gendreau 2005	Milnacipran Placebo	97 28	98% 96%	46.83 48	Yes	12	12
Giordano 1999	Paroxetine Placebo	20 20	100% 100%	31 (overall)	NR	12	12
Goldenberg 1996	Fluoxetine Placebo	15.5 (overall)	90.3% (overall)	43.2 (overall)	No	6	6
Matthey 2013	Milnacipran Placebo	40 40	100% 100%	48.5 50.9	NR	8	7
Mease 2009	Milnacipran Placebo	665 223	95.63% 95.5%	49.44 49.4	Yes	27	27
Murakami 2015	Duloxetine Placebo	196 197	82.2% 84.1%	47.8 49.5	Yes	14	14
Natelson 2015	Milnacipran Placebo	17 17	97.06% (overall)	48 45.6	Yes	8	8
Norregaard 1995	Citalopram Placebo	21 21	NR	48 50	Yes	8	8
Patkar 2007	Paroxetine Placebo	58 58	95% 93%	47.9 49.1	Yes	12	12
Pickering 2018	Milnacipran Placebo	29 25	100% 100%	48 44.3	NR	4	4
Russell 2008	Duloxetine Placebo	376 144	94.71% 95.1%	51.34 50.3	Yes	28	28
Sencan 2004	Paroxetine Placebo	20 20	100% 100%	32.65 35.55	No	6	26
Schmidt-Wilcke 2014	Milnacipran Placebo	11.5 11.5	100% 100%	40.7 (overall)	Yes	6	6
Vitton 2004	Milnacipran Placebo	97 28	NR	NR	Yes	12	12
Wolfe 1994	Fluoxetine Placebo	21 21	100% 100%	48 52.9	No	6	6
Zijlstra 2007	Venlafaxine Placebo	45 45	97.78% 93.33%	47.8 44.8	Yes	6	6

**Table 2:** Measurements for all outcomes across included studies.

**Study ID**	**Intervention**	**Pain (range)**	**Mean (SD) Baseline**	**Mean change (SD)**	**Disability (range)**	**Mean (SD) baseline**	**Mean change (SD)**	**Depression (range)**	**Mean (SD) baseline**	**Mean change (SD)**
Ahmed 2016	Milnacipran Placebo	BPI mean severity score (0–10)	5.4 (1.2) 5.4 (1.2)	−1.3 (2.32)* −0.7 (1.55)*	BPI mean interference score (0–10)	6.4 (1.5) 6.4 (1.5)	2.6 (2.04)* 2.1 (1.76)*	NR	NR NR	NR NR
Allen 2017	Desvenlafaxine Placebo	NRS (0–10)	6.7 (1.29) 6.7 (1.29)	−2.14 (0.23) −2.21 (0.23)	FIQ total score (NR)	NR NR	15.97 (2.95) −15.1 (2.95)	NR	NR NR	NR NR
Anderberg 2000	Citalopram Placebo	VAS pain score (0–10)	5.8 (2) 6.9 (1.4)	−0.71 (0.58) −0.312 (0.58)	NR	NR NR	NR NR	MADRS (1–6)	7.5 (5.9) 7.3 (4.3)	−4.22 (3.46) 0 (3.46)
Arnold 2002	Fluoxetine Placebo	FIQ pain subscore (0–10)	6.1 (1.9) 6 (1.9)	−1.8 (2.4) 0.4 (2.4)	FIQ total score (0–80)	42 (14) 44 (14)	−8.6 (14.5) 2.9 (13.6)	FIQ subscale Depression (0–10)	2.7 (2.7) 2.5 (2)	−0.9 (2.8) 1.1 (2.5)
Arnold 2004	Duloxetine Placebo	FIQ pain subscore (0–10)	6.9 (2.1) 7 (2)	−1.98 (2.96) −1.35 (2.96)	BPI average pain interference (0–10)	5.5 (2.4) 5.5 (2.3)	−2.01 (2.59) −0.95 (2.59)	BDI–II Total score (0–63)	12.7 (9.6) 13.2 (8.9)	−3.32 (7.82) −1.02 (7.82)
Arnold 2005	Duloxetine Placebo	BPI average pain severity (0–10)	6.4 (1.5) 6.5 (1.5)	−2.39 (3.34) −1.16 (2.28)	BPI average pain interference (0–10)	5.9 (2.25) 6 (2.1)	−2.57 (3.34) −1.43 (2.28)	HAMD (0–52)	11.3 (6.3) 11.5 (6.5)	−3.38 (6.69) −2.24 (4.7)
Arnold 2010	Duloxetine Placebo	BPI average pain severity (0–10)	6.5 (1.5) 6.5 (1.6)	−2.3 (2.74) −1.5 (2.82)	BPI average interference (0–10)	6 (2) 6 (2.1)	−2.6 (2.74) −1.7 (2.81)	BDI total score (0–36)	16.2 (10.4) 16.2 (10.4)	−5.5 (8.11) −3.6 (8.17)
Arnold 2010	Milnacipran Placebo	VAS pain score (0–100)	66.8 (16.4) 68.8 (17)	−19.96 (1.57) −12.83 (1.55)	BPI average pain interference (0–10)	NR NR	−1.49 (0.14) −0.91 (0.13)	BDI total score (0–36)	9.1 (6.3) 8.7 (6.5)	−2.12 (0.31) −1.24 (0.31)
Arnold 2012	Duloxetine Placebo	BPI average pain severity (0–10)	6.5 (1.47) 6.37 (1.67)	−2.14 (2.47) −1.86 (2.47)	BPI interference score (0–10)	5.97 (2.17) 5.78 (2.28)	−2.28 (2.47) −1.78 (2.47)	BDI–II total score (0–63)	15 (9.64) 16.84 (11.47)	−5.47 (7.1) −3.91 (7.06)
Branco 2010	Milnacipran Placebo	VAS 24–h recall pain (0–100)	NR NR	−21.9 (25.27) −16.09 (25.27)	BPI SF pain interference (NR)	NR NR	−1.26 (1.98) −0.93 (1.98)	BDI total score (0–36)	10.3 (6.6) 10.9 (6.7)	−0.74 (6.45) −0.29 (6.45)
Chappell 2008	Duloxetine Placebo	FIQ pain score (NR)	NR NR	−1.69 (2.73) −1.06 (2.81)	BPI average interference (0–10)	NR NR	−1.69 (2.51) −1.03 (2.46)	BDI–II Total score (0–63)	NR NR	−3.42 (7.82) −1.45 (7.81)
Clauw 2008	Milnacipran Placebo	Patient experienced pain (0–100)	64.55 (13.65) 65.7 (13.3)	−16.55 (29.54) −13 (29.54)	FIQ total score (NR)	62.1 (13.9) 62.5 (14.1)	−16 (22.71) −12 (22.71)	BDI total score (NR)	13.95 (8.7) 13.8 (9)	−3.3 (8.32) −2.3 (8.32)
Clauw 2013	Milnacipran Placebo	VAS pain score (0–100)	16.6 (9.6) 19.3 (11.6)	8.94 (27.35) 21.3 (27.35)	FIQR total score (0–100)	19.4 (11.9) 21.4 (15.8)	3.78 (16.89) 13.6 (16.89)	SF−36 MCS score (NR)	53.6 (9) 53.6 (11.3)	−2.79 (5.41) −4.64 (5.41)
Gendreau 2005	Milnacipran Placebo	VAS pain score (0–10)	NR NR	−2.26 (3) −0.9 (2.9)	NR	NR NR	NR NR	NR	NR NR	NR NR
Giordano 1999	Paroxetine Placebo	Average score of tender points (1–5)	4.19 (0.35) 3.8 (0.35)	−2.24 (1.91)* 0.3 (1.91)*	NR	NR NR	NR NR	NR	NR NR	NR NR
Goldenberg 1996	Fluoxetine Placebo	VAS pain score (0–100)	68.4 (20.4) 68.4 (20.4)	−10.9 (23.5)* 13.1 (18.76)*	FIQ total score (NR)	57.3 (17.6) 57.3 (17.6)	−9.7 (18.8)* 1.2 (17.36)*	BDI (NR)	12.4 (8.5) 12.4 (8.5)	−4.6 (7.37)* −3.1 (7.7)*
Matthey 2013	Milnacipran Placebo	Current Pain VAS (0–100)	46.8 (18.7) 50.8 (21.8)	−7.2 (21.24)* −2.5 (23.62)*	FIQ total score (0–100)	53.6 (17) 54.7 (14.4)	−9.5 (19.18)*−0.6 (16.9)*	BDI–II Total score (0–63)	10.6 (7.1) 12.6 (7.6)	0.2 (8.84)* 2.4 (8.84)*
Mease 2009	Milnacipran Placebo	VAS 24–h recall pain score (0–100)	73.57 (16.2) 74.3 (15.1)	−30.29 (32.71)* −21.94 (32.81)*	FIQ total score (0–100)	64.57 (14.17) 64.7 (13.4)	−17.41 (18.28) −15.91 (18.28)	NR	NR NR	NR NR
Murakami 2015	Duloxetine Placebo	FIQ pain subscore (NR)	6.83 (1.52) 7.01 (1.67)	−2.37 (4.7) −1.76 (4.89)	BPI interference scores (NR)	5.1 (2.07) 4.95 (2.09)	−1.95 (3.73) −1.44 (3.77)	BDI–II total score (0–63)	15.34 (9.73) 14.89 (9.62)	−4.09 (11.61) −1.19 (11.87)
Natelson 2015	Milnacipran Placebo	VAS pain (NR)	6.43 (1.54) NR	−1.24 (1.57) 0.66 (1.75)	NR	NR NR	NR NR	NR	NR NR	NR NR
Norregaard 1995	Citalopram Placebo	VAS pain (0–10)	6.3 (2) 6.7 (1.9)	−1 (2.1) −0.7 (1.7)	FIQ Physical function (0–3)	1.7 (0.6) 1.7 (0.5)	0 (0.4) 0 (0.4)	BDI (0–36)	16.4 (8.3) 16.3 (8.3)	1 (6.1) 0.9 (7.9)
Patkar 2007	Paroxetine Placebo	VAS pain score (0–100)	74.2 (22.7) 75.3 (19.8)	−12.2 (18.5) −8.8 (16.6)	FIQ total score (0–100)	53 (8.9) 49 (12.2)	−19.7 (13.74) −13.4 (13.74)	NR	NR NR	NR NR
Pickering 2018	Milnacipran Placebo	NRS (0–10)	NR NR	−1 (2.1) −1 (1.7)	NR	NR NR	NR NR	NR	NR NR	NR NR
Russell 2008	Duloxetine Placebo	BPI pain severity score (0–10)	6.52 (1.52) 6.6 (1.7)	−2.14 (4.46) −1.43 (2.52)	FIQ total score (NR)	52.18 (12.67) 53.0 (11.2)	−13.42 (29.67) −10.42 (17.52)	SF-36 mental component (NR)	NR NR	3.73 (20.17) 1.75 (12)
Sencan 2004	Paroxetine Placebo	VAS pain score (0–10)	6.62 (1.42) 7.7 (1.72)	−1.61 (1.72)* −1.86 (1.94)*	NR	NR NR	NR NR	BDI (NR)	20.8 (5.25) 18.5 (5.31)	−10.68 (4.55)* −3.35 (4.63)*
Schmidt–Wilcke 2014	Milnacipran Placebo	BPI sev change score (NR)	NR NR	−0.88 (1.8) −0.17 (2.3)	BPI change score (NR)	NR NR	−1.1 (1.7) −0.56 (2.1)	NR	NR NR	NR NR
Vitton 2004	Milnacipran Placebo	VAS pain score (0–10)	NR NR	−2.3 (3) −0.9 (2.9)	NR	NR NR	NR NR	NR	NR NR	NR NR
Wolfe 1994	Fluoxetine Placebo	VAS pain score (0–3)	1.7 (0.48) 1.8 (0.81)	−0.1 (0.69)* −0.2 (0.8)*	HAQ score (0–3)	0.9 (0.48) 1.1 (0.66)	−0.2 (0.46)*−0.3 (0.72)*	BDI (0–36)	11.8 (7.65) 13.9 (8.86)	−3.5 (6.93)* 0 (9.99)*
Zijlstra 2007	Venlafaxine Placebo	FIQ pain subscore (0–10)	6.4 (1.8) 6.6 (1.6)	−1.3 (2.3) −0.1 (2.1)	FIQ total score (0–80)	44.6 (10) 46.4 (11)	−9 (13) −2.7 (12)	BDI (0–36)	13.6 (6.8) 15.4 (8.2)	−3.4 (4.9) −3.2 (5.8)

*Mean change scores have not been reported in the original study and were thus calculated by the authors.

### Within-Placebo Mean Change Scores of Baseline vs. Follow-Up Assessments

**Figure 2:**
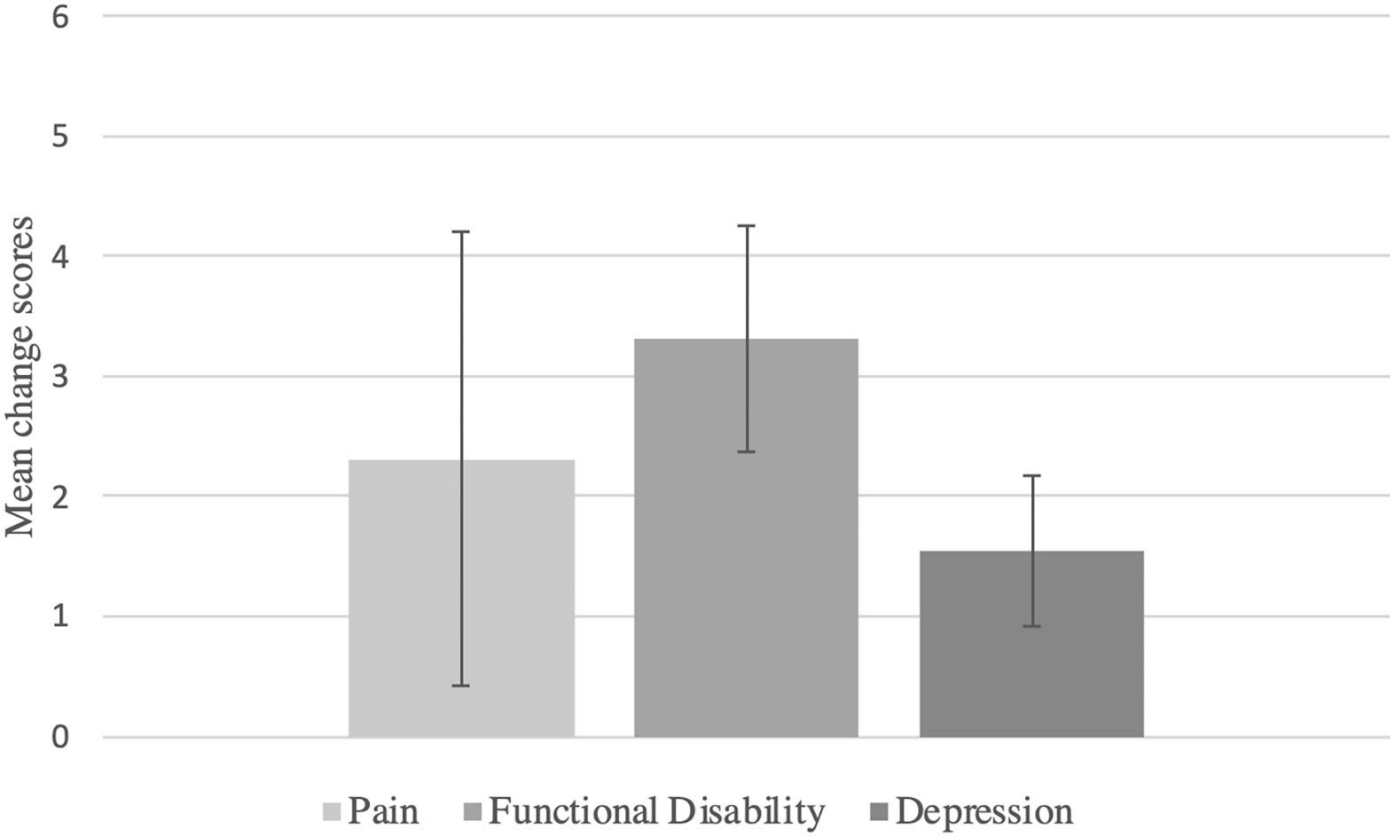
Within-placebo mean change scores of baseline vs. follow-up assessments.

The mean change score for pain reduction in the placebo group was large and statistically significant (mean change score of baseline vs. follow-u*p* = 2.31, 95% CI: 0.42 to 4.21, *p* = 0.017; see Figure 2). Heterogeneity was large with τ^2^ = 25.49, *I*^2^ = 99.9%, and *Q* = 23,728.42 (*p* < 0.000).

For functional disability, the mean reduction was large and statistically significant with a mean change score of baseline vs. follow-up = 3.31, 95% CI: 2.37–4.26, *p* < 0.0001 (see Figure 2). Heterogeneity was found to be large with τ^2^ = 4.02, *I*^2^ = 99.4%, and *Q* = 2,561.9 (*p* < 0.000).

Finally, for depression, the mean change score of baseline vs. follow-up assessment was large and statistically significant again, with a mean change = 1.55, 95% CI: 0.92–2.18, *p* < 0.0001 (see Figure 2). Heterogeneity was large with τ^2^ = 1.32, *I*^2^ = 87.7%, and *Q* = 146.38 (*p* < 0.0001).

## Discussion

The present meta-analysis intended to examine the placebo response in the baseline vs. follow-up comparison in pain, functional disability, and depression in trials examining SSRIs and SNRIs in patients with FM. In total, 29 RCTs were included with a mean treatment duration of 12.5 weeks, which is longer than in previous meta-analyses on antidepressants for FMS (19). We found large and statistically significant within-placebo mean change scores of baseline vs. follow-up assessments.

Notably, the placebo response was highest for the outcome functional disability with a mean change score of baseline vs. follow-up = 3.31 (95% CI: 2.37–4.26, *p* < 0.0001). In the included studies, functional disability measures assessed the impact of FM on a broad range of activities: from mundane everyday tasks, such as self-care and mobility, to general well-being, and engagement in vocational tasks. It has been long-recognized that FM can disrupt these common actions and thus considerably disable patients (49). For many patients maintaining work ability is a primary health concern (50). This is understandable as up to 46% of patients point to their FM as the reason for losing their jobs (51). However, no single treatment option has been established as best to address all the challenges encompassed by functional disability.

Our results indicate that not only a change in pain intensity is possible, but also in other important domains, namely functional disability and depression. These results support the claim that, in many cases, chronic primary pain disorders require a multidisciplinary treatment approach, also referring to the biopsychosocial framework (52–54). Given that placebo responses consists of non-specific effects (besides statistical artifacts and the natural course of the disease), and FM presents as a complex condition, a single-component treatment such as SSRIs and SNRIs falls short (55). From a patients' perspective, however, a reduction in pain intensity is frequently declared to be the most desired treatment outcome (56). Importantly, improvements in different outcome domains do not necessarily correlate with each other, as has been shown in a study that analyzed within-treatment trajectories of patients with chronic pain (57).

Our findings reveal preliminary suggestions for clinical implications. Considering the large placebo response on the different outcome domains, the question arises how these effects can be harnessed in clinical practice. First of all, it is important to clearly define what a placebo is. In research, placebos in randomized controlled trials are used to control for confounders associated with clinical trials, such as spontaneous remission and regression toward the mean (58). In clinical practice, however, placebos can be utilized to enhance positive outcomes by means of well-known placebo mechanisms. These include positive treatment expectations, a patient-physician relationship that is built on trust, and a plausible treatment narrative (59). With the aim to actively harness these mechanisms, the following suggestions might be taken into account when treating patients living with FM: (1) to address key ethical principles such as autonomy and transparency during the administration of SSRIs and SNRIs, i.e., by talking about the empirical evidence for the intervention, including placebo responses and their underlying processes (60); (2) to foster a patient-physician relationship that is based on trust, i.e., by ensuring that patients feel understood and cared for (61); and (3) to address and discuss patients' expectations, i.e., by asking what they expect about the treatment, what wishes and fears are associated with the prospect of receiving SSRIs and SNRIs (62).

Two additional approaches that have been studied in the past and enable to harness placebo effects in the clinical practice are the following: First, placebos could be used as dose extenders. By pairing placebo pills with a physiologically active drug, studies have revealed that medication dosages can be substantially lowered without decreasing the efficacy of the drug (63, 64). A second strategy is known as open-label placebo administration, i.e., the placebo treatment with full disclosure. Open-label placebos are administered with a scientific rationale, i.e., patients are told that ‘we know that placebos have powerful effects' (65). Two meta-analyses reveal that the open-label placebo therapy shows statistically significant and clinically meaningful effects in pain and non-pain conditions (66, 67).

Our analysis has several limitations. First and foremost, within-group analyses have limited validity (68): Mean change scores of baseline vs. follow-up assessments are not independent of each other, since baseline and follow-up scores are correlated. Furthermore, they are affected the natural course and characteristics of the patients and settings, and these cannot be disentangled from the effects of the intervention. However, we were especially interested to research preliminary indication for the potential of placebo in this population and to focus on first recommendations for the clinical routine. Second, since included studies span more than two decades, it cannot be ruled out that a change in the diagnostic criteria over time may have influenced the findings. Third, due to small sample sizes in some SSRI/SNRI treatments, these results might be statistically underpowered. Therefore, some effects might be due to the so-called small-study effect. This means that smaller trials show different, sometimes larger, treatment effects than bigger studies (69). Fourth, treatment duration of included interventions varied largely between 4 and 28 weeks. The optimal duration of treatment therefore remains unclear, and the short duration of several studies leads to open questions with regard to long-term beneficial effects of SSRI/SNRI treatments on FMS symptoms. In a similar fashion, the time points for follow-up assessments varied, which might have contributed to heterogeneity in our results. Finally, the systematic literature search was conducted 2 years ago, hence we cannot rule out that the inclusion of newer studies would have changed the results of our analyses.

Future studies should have an in-depth examination of the placebo response by using individual patient data instead of aggregate data. This would allow to determine patient-related and trial-related placebo moderators and would therefore be in line with the personalized medicine approach (70). This is also strengthened by our data that showed substantial heterogeneity across outcomes. Furthermore, and in order to disentangle placebo effects from the natural course and statistical artifacts, it would be advantageable to compare a placebo arm with a no-treatment arm in SSRI and SNRI trials (25).

In conclusion, our results provide preliminary evidence that placebo responses, which also consist of non-specific effects, might play a role in the treatment of FM.

## Data Availability Statement

The original contributions presented in the study are included in the article, further inquiries can be directed to the corresponding author/s.

## Author Contributions

HK and CL initiated the study concept, conducted the analyses, and drafted the paper. HK, CL, and AK designed the extraction template. TP and JP extracted the data. HK, CL, TP, JP, AK, and SB wrote the final paper, critically revised the manuscript, and gave important intellectual contribution to it. All authors have read and approved the manuscript.

## Funding

CL received funding from the Swiss National Science Foundation (SNSF): P4P4PS_194536. HK is sponsored by the Swiss National Science Foundation (SNSF) fellowship P400PS_186658. AK received funding from the EPIC (eHealth Productivity and Innovation in Cornwall and the Isles of Scilly) project, which was in part funded by the University of Plymouth and the European Regional Development Fund (ERDF), Grant Number [05R18P02814]. The funding bodies have no role in the design of the study and collection, analysis, and interpretation of data and manuscript writing.

## Conflict of Interest

The authors declare that the research was conducted in the absence of any commercial or financial relationships that could be construed as a potential conflict of interest.

## Publisher's Note

All claims expressed in this article are solely those of the authors and do not necessarily represent those of their affiliated organizations, or those of the publisher, the editors and the reviewers. Any product that may be evaluated in this article, or claim that may be made by its manufacturer, is not guaranteed or endorsed by the publisher.
