# Supplementary material for: Placebo Responses and Their Clinical Implications in Fibromyalgia: A Meta-Analysis Using SSRI and SNRI Trials
Source: Front Pain Res (Lausanne). 2021 Dec 7;2:750523. doi: 10.3389/fpain.2021.750523 (PMC8915657; doi:10.3389/fpain.2021.750523)
Supplement: Supplementary file 1 [file Data_Sheet_1.PDF]

BEST TREATMENT OPTION(S) FOR PATIENTS WITH CHRONIC PRIMARY PAIN: A NETWORK META-ANALYTIC APPROACH SEARCH METHODS

Database Searched	Date Searched	Results Returned
**MEDLINE**	05/04/2018	18,963
**Embase**	06/04/2018	32,674
**CENTRAL**	06/04/2018	13,536
**PsycINFO**	06/04/2018	4,851
**Web of Science**	06/04/2018	21,006
---	---	---
**Database Total**	**---**	**91,030**
**Clinicaltrials.gov**	06/04/2018	3,243
**OpenTrials (includes WHO ICTRP, GlaxoSmithKline, ISRCTN, EU Clinical Trial Register, Clinicaltrials.gov)**	09/04/2018	2,849
**---**	---	---
**Grey Literature Search Total**	**---**	**6,092**
**---**	---	---
Total All	---	97,122
Duplicates Removed	---	37,709
**Total after Duplicates Removed**	**---**	**59,413**

MEDLINE

Database: Ovid MEDLINE(R) Epub Ahead of Print, In-Process & Other Non-Indexed Citations, Ovid MEDLINE(R) Daily and Ovid MEDLINE(R) <1946 to Present>

Search Strategy:

----

1 Chronic Pain/dh, dt, rh, th [Diet Therapy, Drug Therapy, Rehabilitation, Therapy] (5177)

2 ((chronic adj4 pain?) and (drug? or pharma* or treat* or therap* or management or intervention? or reduce or reduction or counselling or exercise or effect or effectiveness or efficacy)).tw,kw. (39795)

3 Fibromyalgia/dh, dt, rh, th (2880)

4 ((fibromyalgia? or fibrositis or fibrositic nodule) and (drug? or pharma* or treat* or therap* or management or intervention? or reduce or reduction or counselling or exercise or effect or effectiveness or efficacy)).tw,kw. (5379)

5 complex regional pain syndromes/dh, dt, rh, th (584)

6 ((crps or ((regional complex or complex regional) adj3 pain)) and (drug? or pharma* or treat* or therap* or management or intervention? or reduce or reduction or counselling or exercise or effect or effectiveness or efficacy)).tw,kw. (2034)

7 causalgia/dh, dt, rh, th (247)

8 ((causalgia or (deafferentation adj pain)) and (drug? or pharma* or treat* or therap* or management or intervention? or reduce or reduction or counselling or exercise or effect or effectiveness or efficacy)).tw,kw. (501)

9 reflex sympathetic dystrophy/dh, dt, rh, th (1301)

10 ((algesidystrophy or algodystrophia or algoneurodystrophy or (sudek* adj2 (atroph* or dystrophy or disease or syndrome)) or (cervical sympathetic adj2 dystroph*) or rsd? or (reflex sympathetic adj2 dystroph*) or ((shoulder arm or shoulder hand) adj syndrome)) and (drug? or pharma* or treat* or therap* or management or intervention? or reduce or reduction or counselling or exercise or effect or effectiveness or efficacy)).tw,kw. (7915)

11 (((neuralgic shoulder adj amyotophy) or ((post traumatic or posttraumatic or reflex sympathetic) adj dystrophy) or autonomic neuropathy or sympathetic dystrophy or (posttraumatic adj (osteoporosis or osteopenia))) and (drug? or pharma* or treat* or therap* or management or intervention? or reduce or reduction or counselling or exercise or effect or effectiveness or efficacy)).tw,kw. (2828)

12 ((chronic adj2 primary headache) and (drug? or pharma* or treat* or therap* or management or intervention? or reduce or reduction or counselling or exercise or effect or effectiveness or efficacy)).tw,kw. (27)

13 facial pain/dh, dt, rh, th (1617)

14 ((chronic adj3 (orofacial pain or mandibular pain or maxillary pain or jaw pain or face pain or facial neuralgia or facial pain or facialgia)) and (drug? or pharma* or treat* or therap* or management or intervention? or reduce or reduction or counselling or exercise or effect or effectiveness or efficacy)).tw,kw. (438)

15 migraine disorders/dh, dt, rh, th (9412)

16 ((migraine? or hemicrania) and (drug? or pharma* or treat* or therap* or management or intervention? or reduce or reduction or counselling or exercise or effect or effectiveness or efficacy)).tw,kw. (17328)

17 migraine with aura/dh, dt, rh, th (261)

18 migraine without aura/dh, dt, rh, th (163)

19 ophthalmoplegic migraine/dh, dt, rh, th (2)

20 Tension-Type Headache/dh, dt, rh, th [Diet Therapy, Drug Therapy, Rehabilitation, Therapy] (723)

21 (((tension* or idiopathic or psychogenic or stress) adj headache?) and (drug? or pharma* or treat* or therap* or management or intervention? or reduce or reduction or counselling or exercise or effect or effectiveness or efficacy)).tw,kw. (754)

22 trigeminal autonomic cephalalgias/dh, dt, rh, th (87)

23 ((trigeminal autonomic adj cephal*) and (drug? or pharma* or treat* or therap* or management or intervention? or reduce or reduction or counselling or exercise or effect or effectiveness or efficacy)).tw,kw. (230)

24 cluster headache/dh, dt, rh, th (1003)

25 (((cluster adj2 headache?) or histamine cephalgia? or horton* syndrome or ciliary neuralgia?) and (drug? or pharma* or treat* or therap* or management or intervention? or reduce or reduction or counselling or exercise or effect or effectiveness or efficacy)).tw,kw. (1595)

26 paroxysmal hemicrania/dh, dt, rh, th (60)

27 (paroxysmal hemicrania and (drug? or pharma* or treat* or therap* or management or intervention? or reduce or reduction or counselling or exercise or effect or effectiveness or efficacy)).tw,kw. (214)

28 sunct syndrome/dh, dt, rh, th (65)

29 ((sunct adj syndrome?) and (drug? or pharma* or treat* or therap* or management or intervention? or reduce or reduction or counselling or exercise or effect or effectiveness or efficacy)).tw,kw. (106)

30 temporomandibular joint disorders/dh, dt, rh, th (2744)

31 temporomandibular joint dysfunction syndrome/dh, dt, rh, th (1714)

32 (((temporomandibular or TMJ) adj5 (disease? or syndrome)) and (drug? or pharma* or treat* or therap* or management or intervention? or reduce or reduction or counselling or exercise or effect or effectiveness or efficacy)).tw,kw. (777)

33 (costen* syndrome and (drug? or pharma* or treat* or therap* or management or intervention? or reduce or reduction or counselling or exercise or effect or effectiveness or efficacy)).tw,kw. (17)

34 Burning Mouth Syndrome/dt, th [Drug Therapy, Therapy] (309)

35 (((burning mouth adj syndrome) or oral dys?esthesia) and (drug? or pharma* or treat* or therap* or management or intervention? or reduce or reduction or counselling or exercise or effect or effectiveness or efficacy)).tw,kw. (471)

36 Visceral Pain/dh, dt, rh, th [Diet Therapy, Drug Therapy, Rehabilitation, Therapy] (144)

37 chest pain/dh, dt, rh, th (1273)

38 ((primary adj2 chest pain) and (drug? or pharma* or treat* or therap* or management or intervention? or reduce or reduction or counselling or exercise or effect or effectiveness or efficacy)).tw,kw. (29)

39 ((Epigastric pain adj syndrome) and (drug? or pharma* or treat* or therap* or management or intervention? or reduce or reduction or counselling or exercise or effect or effectiveness or efficacy)).tw,kw. (76)

40 Irritable Bowel Syndrome/dh, dt, rh, th [Diet Therapy, Drug Therapy, Rehabilitation, Therapy] (2467)

41 Colonic Diseases, Functional/dh, dt, rh, th (1397)

42 limit 41 to yr="1970 - 2003" (1294)

43 Colonic Diseases/ (15485)

44 limit 43 to yr="1967 - 1970" (864)

45 ((IBS or (mucous adj coliti*) or (irritable adj (bowel or colon))) and (drug? or pharma* or treat* or therap* or management or intervention? or reduce or reduction or counselling or exercise or effect or effectiveness or efficacy)).tw,kw. (7409)

46 Abdominal Pain/dh, dt, rh, th [Diet Therapy, Drug Therapy, Rehabilitation, Therapy] (1894)

47 ((bladder pain adj2 syndrome) and (drug? or pharma* or treat* or therap* or management or intervention? or reduce or reduction or counselling or exercise or effect or effectiveness or efficacy)).tw,kw. (390)

48 pelvic pain/dh, dt, rh, th (1339)

49 pelvic girdle pain/dh, dt, rh, th (33)

50 ((chronic adj4 pelvic pain) and (drug? or pharma* or treat* or therap* or management or intervention? or reduce or reduction or counselling or exercise or effect or effectiveness or efficacy)).tw,kw. (2336)

51 Low Back Pain/dt, rh, th [Drug Therapy, Rehabilitation, Therapy] (7852)

52 Neck Pain/dh, dt, rh, th (1975)

53 or/1-40,42,44-52 (104031)

54 Randomized Controlled Trial.pt. (456937)

55 Pragmatic Clinical Trial.pt. (709)

56 exp Randomized Controlled Trials as Topic/ (117086)

57 "Randomized Controlled Trial (topic)"/ (0)

58 Randomized Controlled Trial/ (456937)

59 Randomization/ (93683)

60 Random Allocation/ (93683)

61 Double-Blind Method/ (144874)

62 Double Blind Procedure/ (0)

63 Double-Blind Studies/ (144874)

64 Single-Blind Method/ (24871)

65 Single Blind Procedure/ (0)

66 Single-Blind Studies/ (24871)

67 Placebos/ (33843)

68 Placebo/ (0)

69 (random* or sham or placebo*).ti,ab,hw,kf,kw. (1296751)

70 ((singl* or doubl*) adj (blind* or dumm* or mask*)).ti,ab,hw,kf,kw. (213376)

71 ((tripl* or trebl*) adj (blind* or dumm* or mask*)).ti,ab,hw,kf,kw. (711)

72 or/54-71 (1321075)

73 53 and 72 (18963)

****

Embase

Database: Embase <1974 to 2018 April 05>

Search Strategy:

----

1 chronic pain/dt, rh, th [Drug Therapy, Rehabilitation, Therapy] (16728)

2 ((chronic adj4 pain?) and (drug? or pharma* or treat* or therap* or management or intervention? or reduce or reduction or counselling or exercise or effect or effectiveness or efficacy)).tw,kw. (62018)

3 fibromyalgia/dt, rh, th (4950)

4 ((fibromyalgia? or fibrositis or fibrositic nodule) and (drug? or pharma* or treat* or therap* or management or intervention? or reduce or reduction or counselling or exercise or effect or effectiveness or efficacy)).tw,kw. (9050)

5 complex regional pain syndrome/dt, rh, th [Drug Therapy, Rehabilitation, Therapy] (1220)

6 ((crps or ((regional complex or complex regional) adj3 pain)) and (drug? or pharma* or treat* or therap* or management or intervention? or reduce or reduction or counselling or exercise or effect or effectiveness or efficacy)).tw,kw. (3457)

7 complex regional pain syndrome type II/dt, rh, th [Drug Therapy, Rehabilitation, Therapy] (98)

8 ((causalgia or (deafferentation adj pain)) and (drug? or pharma* or treat* or therap* or management or intervention? or reduce or reduction or counselling or exercise or effect or effectiveness or efficacy)).tw,kw. (698)

9 complex regional pain syndrome type i/dt, rh, th or algodystrophy/dt, rh, th or posttraumatic osteoporosis/ or sympathetic dystrophy/dt, rh, th (1954)

10 ((algesidystrophy or algodystrophia or algoneurodystrophy or (sudek* adj2 (atroph* or dystrophy or disease or syndrome)) or (cervical sympathetic adj2 dystroph*) or rsd? or (reflex sympathetic adj2 dystroph*) or ((shoulder arm or shoulder hand) adj syndrome)) and (drug? or pharma* or treat* or therap* or management or intervention? or reduce or reduction or counselling or exercise or effect or effectiveness or efficacy)).tw,kw. (11486)

11 (((neuralgic shoulder adj amyotophy) or ((post traumatic or posttraumatic or reflex sympathetic) adj dystrophy) or autonomic neuropathy or sympathetic dystrophy or (posttraumatic adj (osteoporosis or osteopenia))) and (drug? or pharma* or treat* or therap* or management or intervention? or reduce or reduction or counselling or exercise or effect or effectiveness or efficacy)).tw,kw. (4460)

12 primary headache/dt, th [Drug Therapy, Therapy] (265)

13 ((chronic adj2 primary headache) and (drug? or pharma* or treat* or therap* or management or intervention? or reduce or reduction or counselling or exercise or effect or effectiveness or efficacy)).tw,kw. (48)

14 face pain/dt, rh, th [Drug Therapy, Rehabilitation, Therapy] (2019)

15 jaw pain/dt, th [Drug Therapy, Therapy] (70)

16 ((chronic adj3 (orofacial pain or mandibular pain or maxillary pain or jaw pain or face pain or facial neuralgia or facial pain or facialgia)) and (drug? or pharma* or treat* or therap* or management or intervention? or reduce or reduction or counselling or exercise or effect or effectiveness or efficacy)).tw,kw. (519)

17 migraine/dt, rh, th [Drug Therapy, Rehabilitation, Therapy] (15314)

18 ((migraine? or hemicrania) and (drug? or pharma* or treat* or therap* or management or intervention? or reduce or reduction or counselling or exercise or effect or effectiveness or efficacy)).tw,kw. (28195)

19 basilar type migraine/dt, rh, th or complicated migraine/dt, rh, th or episodic migraine/dt, rh, th or menstrual migraine/dt, rh, th or migraine aura/dt, rh, th or migraine with aura/dt, rh, th or migraine without

aura/dt, rh, th or migrainous infarction/dt, rh, th or ophthalmoplegic migraine/dt, rh, th or retinal migraine/dt, rh, th or transformed migraine/dt, rh, th or vestibular migraine/dt, rh, th (2209)

20 hemiplegic migraine/dt, rh, th or familial hemiplegic migraine/dt, rh, th or sporadic hemiplegic migraine/dt, rh, th (139)

21 chronic tension headache/dt, rh, th [Drug Therapy, Rehabilitation, Therapy] (248)

22 (((tension* or idiopathic or psychogenic or stress) adj headache?) and (drug? or pharma* or treat* or therap* or management or intervention? or reduce or reduction or counselling or exercise or effect or effectiveness or efficacy)).tw,kw. (1255)

23 trigeminal autonomic cephalalgia/dt, th [Drug Therapy, Therapy] (114)

24 cluster headache/dt, rh, th or chronic cluster headache/dt, rh, th (1874)

25 (((cluster adj2 headache?) or histamine cephalgia? or horton* syndrome or ciliary neuralgia?) and (drug? or pharma* or treat* or therap* or management or intervention? or reduce or reduction or counselling or exercise or effect or effectiveness or efficacy)).tw,kw. (2650)

26 paroxysmal hemicrania/dt, rh, th or chronic paroxysmal hemicrania/dt, rh, th (158)

27 (paroxysmal hemicrania and (drug? or pharma* or treat* or therap* or management or intervention? or reduce or reduction or counselling or exercise or effect or effectiveness or efficacy)).tw,kw. (305)

28 SUNCT syndrome/dt, th [Drug Therapy, Therapy] (214)

29 ((sunct adj syndrome?) and (drug? or pharma* or treat* or therap* or management or intervention? or reduce or reduction or counselling or exercise or effect or effectiveness or efficacy)).tw,kw. (138)

30 temporomandibular joint disorder/dt, rh, th [Drug Therapy, Rehabilitation, Therapy] (2814)

31 (((temporomandibular or TMJ) adj5 (disease? or syndrome)) and (drug? or pharma* or treat* or therap* or management or intervention? or reduce or reduction or counselling or exercise or effect or effectiveness or efficacy)).tw,kw. (958)

32 (costen* syndrome and (drug? or pharma* or treat* or therap* or management or intervention? or reduce or reduction or counselling or exercise or effect or effectiveness or efficacy)).tw,kw. (22)

33 burning mouth syndrome/dt, th [Drug Therapy, Therapy] (405)

34 (((burning mouth adj syndrome) or oral dys?esthesia) and (drug? or pharma* or treat* or therap* or management or intervention? or reduce or reduction or counselling or exercise or effect or effectiveness or efficacy)).tw,kw. (634)

35 visceral pain/dt, th [Drug Therapy, Therapy] (924)

36 thorax pain/dt, rh, th [Drug Therapy, Rehabilitation, Therapy] (3159)

37 ((primary adj2 chest pain) and (drug? or pharma* or treat* or therap* or management or intervention? or reduce or reduction or counselling or exercise or effect or effectiveness or efficacy)).tw,kw. (46)

38 ((Epigastric pain adj syndrome) and (drug? or pharma* or treat* or therap* or management or intervention? or reduce or reduction or counselling or exercise or effect or effectiveness or efficacy)).tw,kw. (175)

39 irritable colon/dt, rh, th [Drug Therapy, Rehabilitation, Therapy] (5547)

40 ((IBS or (mucous adj coliti*) or (irritable adj (bowel or colon))) and (drug? or pharma* or treat* or therap* or management or intervention? or reduce or reduction or counselling or exercise or effect or effectiveness or efficacy)).tw,kw. (13284)

41 abdominal pain/dt, rh, th or abdominal angina/dt, rh, th or lower abdominal pain/dt, rh, th or upper abdominal pain/dt, rh, th (5229)

42 ((bladder pain adj2 syndrome) and (drug? or pharma* or treat* or therap* or management or intervention? or reduce or reduction or counselling or exercise or effect or effectiveness or efficacy)).tw,kw. (747)

43 pelvic pain/dt, rh, th or pelvis pain syndrome/dt, rh, th (2352)

44 ((chronic adj4 pelvic pain) and (drug? or pharma* or treat* or therap* or management or intervention? or reduce or reduction or counselling or exercise or effect or effectiveness or efficacy)).tw,kw. (4031)

45 low back pain/dt, rh, th [Drug Therapy, Rehabilitation, Therapy] (12930)

46 neck pain/dt, rh, th [Drug Therapy, Rehabilitation, Therapy] (3293)

47 or/1-46 (168532)

48 Randomized Controlled Trial.pt. (0)

49 Pragmatic Clinical Trial.pt. (0)

50 exp Randomized Controlled Trials as Topic/ (143070)

51 "Randomized Controlled Trial (topic)"/ (143070)

52 Randomized Controlled Trial/ (496986)

53 Randomization/ (77634)

54 Random Allocation/ (74440)

55 Double-Blind Method/ (125684)

56 Double Blind Procedure/ (148540)

57 Double-Blind Studies/ (109931)

58 Single-Blind Method/ (29580)

59 Single Blind Procedure/ (30977)

60 Single-Blind Studies/ (30977)

61 Placebos/ (266819)

62 Placebo/ (323132)

63 (random* or sham or placebo*).ti,ab,hw,kf,kw. (1759202)

64 ((singl* or doubl*) adj (blind* or dumm* or mask*)).ti,ab,hw,kf,kw. (270964)

65 ((tripl* or trebl*) adj (blind* or dumm* or mask*)).ti,ab,hw,kf,kw. (939)

66 or/48-65 (1790065)

67 47 and 66 (32674)

****

CENTRAL

Database: EBM Reviews - Cochrane Central Register of Controlled Trials <March 2018>
Search Strategy:
----
1 Chronic Pain/dh, dt, rh, th [Diet Therapy, Drug Therapy, Rehabilitation, Therapy] (33)
2 ((chronic adj4 pain?) and (drug? or pharma* or treat* or therap* or management or intervention? or reduce or reduction or counselling or exercise or effect or effectiveness or efficacy)).tw,kw. (7750)
3 Fibromyalgia/dh, dt, rh, th (85)
4 ((fibromyalgia? or fibrositis or fibrositic nodule) and (drug? or pharma* or treat* or therap* or management or intervention? or reduce or reduction or counselling or exercise or effect or effectiveness or efficacy)).tw,kw. (1387)
5 complex regional pain syndromes/dh, dt, rh, th (44)
6 ((crps or ((regional complex or complex regional) adj3 pain)) and (drug? or pharma* or treat* or therap* or management or intervention? or reduce or reduction or counselling or exercise or effect or effectiveness or efficacy)).tw,kw. (253)
7 causalgia/dh, dt, rh, th (3)
8 ((causalgia or (deafferentation adj pain)) and (drug? or pharma* or treat* or therap* or management or intervention? or reduce or reduction or counselling or exercise or effect or effectiveness or efficacy)).tw,kw. (23)
9 reflex sympathetic dystrophy/dh, dt, rh, th (61)
10 ((algesidystrophy or algodystrophia or algoneurodystrophy or (sudek* adj2 (atroph* or dystrophy or disease or syndrome)) or (cervical sympathetic adj2 dystroph*) or rsd? or (reflex sympathetic adj2 dystroph*) or ((shoulder arm or shoulder hand) adj syndrome)) and (drug? or pharma* or treat* or therap* or management or intervention? or reduce or reduction or counselling or exercise or effect or effectiveness or efficacy)).tw,kw. (262)
11 (((neuralgic shoulder adj amyotophy) or ((post traumatic or posttraumatic or reflex sympathetic) adj dystrophy) or autonomic neuropathy or sympathetic dystrophy or (posttraumatic adj (osteoporosis or osteopenia))) and (drug? or pharma* or treat* or therap* or management or intervention? or reduce or reduction or counselling or exercise or effect or effectiveness or efficacy)).tw,kw. (321)
12 ((chronic adj2 primary headache) and (drug? or pharma* or treat* or therap* or management or intervention? or reduce or reduction or counselling or exercise or effect or effectiveness or efficacy)).tw,kw. (8)

13 facial pain/dh, dt, rh, th (21)

14 ((chronic adj3 (orofacial pain or mandibular pain or maxillary pain or jaw pain or face pain or facial neuralgia or facial pain or facialgia)) and (drug? or pharma* or treat* or therap* or management or intervention? or reduce or reduction or counselling or exercise or effect or effectiveness or efficacy)).tw,kw. (50)

15 migraine disorders/dh, dt, rh, th (139)

16 ((migraine? or hemicrania) and (drug? or pharma* or treat* or therap* or management or intervention? or reduce or reduction or counselling or exercise or effect or effectiveness or efficacy)).tw,kw. (3258)

17 migraine with aura/dh, dt, rh, th (29)

18 migraine without aura/dh, dt, rh, th (15)

19 ophthalmoplegic migraine/dh, dt, rh, th (0)

20 Tension-Type Headache/dh, dt, rh, th [Diet Therapy, Drug Therapy, Rehabilitation, Therapy] (127)

21 (((tension* or idiopathic or psychogenic or stress) adj headache?) and (drug? or pharma* or treat* or therap* or management or intervention? or reduce or reduction or counselling or exercise or effect or effectiveness or efficacy)).tw,kw. (356)

22 trigeminal autonomic cephalalgias/dh, dt, rh, th (1)

23 ((trigeminal autonomic adj cephal*) and (drug? or pharma* or treat* or therap* or management or intervention? or reduce or reduction or counselling or exercise or effect or effectiveness or efficacy)).tw,kw. (4)

24 cluster headache/dh, dt, rh, th (18)

25 (((cluster adj2 headache?) or histamine cephalgia? or horton* syndrome or ciliary neuralgia?) and (drug? or pharma* or treat* or therap* or management or intervention? or reduce or reduction or counselling or exercise or effect or effectiveness or efficacy)).tw,kw. (149)

26 paroxysmal hemicrania/dh, dt, rh, th (0)

27 (paroxysmal hemicrania and (drug? or pharma* or treat* or therap* or management or intervention? or reduce or reduction or counselling or exercise or effect or effectiveness or efficacy)).tw,kw. (0)

28 sunct syndrome/dh, dt, rh, th (0)

29 ((sunct adj syndrome?) and (drug? or pharma* or treat* or therap* or management or intervention? or reduce or reduction or counselling or exercise or effect or effectiveness or efficacy)).tw,kw. (1)

30 temporomandibular joint disorders/dh, dt, rh, th (174)

31 temporomandibular joint dysfunction syndrome/dh, dt, rh, th (82)

32 (((temporomandibular or TMJ) adj5 (disease? or syndrome)) and (drug? or pharma* or treat* or therap* or management or intervention? or reduce or reduction or counselling or exercise or effect or effectiveness or efficacy)).tw,kw. (61)

33 (costen* syndrome and (drug? or pharma* or treat* or therap* or management or intervention? or reduce or reduction or counselling or exercise or effect or effectiveness or efficacy)).tw,kw. (0)

34 Burning Mouth Syndrome/dt, th [Drug Therapy, Therapy] (34)

35 (((burning mouth adj syndrome) or oral dys?esthesia) and (drug? or pharma* or treat* or therap* or management or intervention? or reduce or reduction or counselling or exercise or effect or effectiveness or efficacy)).tw,kw. (85)

36 Visceral Pain/dh, dt, rh, th [Diet Therapy, Drug Therapy, Rehabilitation, Therapy] (3)

37 chest pain/dh, dt, rh, th (7)
38 ((primary adj2 chest pain) and (drug? or pharma* or treat* or therap* or management or intervention? or reduce or reduction or counselling or exercise or effect or effectiveness or efficacy)).tw,kw. (15)
39 ((Epigastric pain adj syndrome) and (drug? or pharma* or treat* or therap* or management or intervention? or reduce or reduction or counselling or exercise or effect or effectiveness or efficacy)).tw,kw. (28)
40 Irritable Bowel Syndrome/dh, dt, rh, th [Diet Therapy, Drug Therapy, Rehabilitation, Therapy] (207)
41 Colonic Diseases, Functional/dh, dt, rh, th (157)
42 limit 41 to yr="1970 - 2003" (146)
43 Colonic Diseases/ (282)
44 limit 43 to yr="1967 - 1970" (5)
45 ((IBS or (mucous adj coliti*) or (irritable adj (bowel or colon))) and (drug? or pharma* or treat* or therap* or management or intervention? or reduce or reduction or counselling or exercise or effect or effectiveness or efficacy)).tw,kw. (2054)
46 Abdominal Pain/dh, dt, rh, th [Diet Therapy, Drug Therapy, Rehabilitation, Therapy] (24)
47 ((bladder pain adj2 syndrome) and (drug? or pharma* or treat* or therap* or management or intervention? or reduce or reduction or counselling or exercise or effect or effectiveness or efficacy)).tw,kw. (73)
48 pelvic pain/dh, dt, rh, th (20)
49 pelvic girdle pain/dh, dt, rh, th (8)
50 ((chronic adj4 pelvic pain) and (drug? or pharma* or treat* or therap* or management or intervention? or reduce or reduction or counselling or exercise or effect or effectiveness or efficacy)).tw,kw. (366)
51 Low Back Pain/dt, rh, th [Drug Therapy, Rehabilitation, Therapy] (184)
52 Neck Pain/dh, dt, rh, th (41)
53 or/1-40,42,44-52 (15736)
54 Randomized Controlled Trial.pt. (446483)
55 Pragmatic Clinical Trial.pt. (698)
56 exp Randomized Controlled Trials as Topic/ (7376)
57 "Randomized Controlled Trial (topic)"/ (7)
58 Randomized Controlled Trial/ (139)
59 Randomization/ (3)
60 Random Allocation/ (20579)
61 Double-Blind Method/ (125619)
62 Double Blind Procedure/ (16)
63 Double-Blind Studies/ (1)
64 Single-Blind Method/ (17667)
65 Single Blind Procedure/ (1)
66 Single-Blind Studies/ (17667)
67 Placebos/ (23173)
68 Placebo/ (32)
69 (random* or sham or placebo*).ti,ab,hw,kf,kw. (696661)
70 ((singl* or doubl*) adj (blind* or dumm* or mask*)).ti,ab,hw,kf,kw. (252338)

71 ((tripl* or trebl*) adj (blind* or dumm* or mask*)).ti,ab,hw,kf,kw. (939)
72 or/54-71 (798371)
73 53 and 72 (13536)

****

PsycINFO

#	Query	Results
S34	S29 AND S33	4,851
S33	S30 OR S31 OR S32	222,208
S32	((singl* N1 blind*) or (doubl* N1 blind*) or (tripl* N1 blind*) or (trebl* N1 blind*)) OR ((singl* N1 dumm*) or (doubl* N1 dumm*) or (tripl* N1 dumm*) or (trebl* N1 dumm*)) OR ((singl* N1 mask*) or (doubl* N1 mask*) or (tripl* N1 mask*) or (trebl* N1 mask*))	31,406
S31	(random* or sham or placebo*)	216,631
S30	(DE "Placebo") OR (DE "Random Sampling")	5,848
S29	S1 OR S2 OR S3 OR S4 OR S5 OR S6 OR S7 OR S8 OR S9 OR S10 OR S11 OR S12 OR S13 OR S14 OR S15 OR S16 OR S17 OR S18 OR S19 OR S20 OR S21 OR S22 OR S23 OR S24 OR S25 OR S26 OR S27 OR S28	29,567
S28	(chronic N4 pelvic pain) AND (drug? or pharma* or treat* or therap* or management or intervention? or reduce or reduction or counselling or exercise or effect or effectiveness or efficacy)	243
S27	(bladder pain N2 syndrome) AND (drug? or pharma* or treat* or therap* or management or intervention? or reduce or reduction or counselling or exercise or effect or effectiveness or efficacy)	24
S26	((IBS or (mucous N1 coliti*) or (irritable N1 bowel) or (irritable N1 colon))) AND (drug? or pharma* or treat* or therap* or management or intervention? or reduce or reduction or counselling or exercise or effect or effectiveness or efficacy)	1,457
S25	(DE "Irritable Bowel Syndrome") AND (drug# or pharma* or treat* or therap* or management or intervention# or reduce or reduction or counselling or exercise or effect or effectiveness or efficacy)	856
S24	(Epigastric pain N1 syndrome) AND (drug? or pharma* or treat* or therap* or management or intervention? or reduce or reduction or counselling or exercise or effect or effectiveness or efficacy)	1

S23	(primary N2 chest pain) AND (drug# or pharma* or treat* or therap* or management or intervention# or reduce or reduction or counselling or exercise or effect or effectiveness or efficacy)	10
S22	(((burning mouth N1 syndrome) or oral dys#esthesia)) AND (drug# or pharma* or treat* or therap* or management or intervention# or reduce or reduction or counselling or exercise or effect or effectiveness or efficacy)	83
S21	(costen* syndrome) AND (drug# or pharma* or treat* or therap* or management or intervention# or reduce or reduction or counselling or exercise or effect or effectiveness or efficacy)	1
S20	((temporomandibular N5 disease#) or (temporomandibular N5 syndrome) or (TMJ N5 disease#) or (TMJ N5 syndrome)) AND (drug# or pharma* or treat* or therap* or management or intervention# or reduce or reduction or counselling or exercise or effect or effectiveness or efficacy)	113
S19	(sunct N1 syndrome#) AND (drug# or pharma* or treat* or therap* or management or intervention# or reduce or reduction or counselling or exercise or effect or effectiveness or efficacy)	76
S18	(paroxysmal hemicrania) AND (drug# or pharma* or treat* or therap* or management or intervention# or reduce or reduction or counselling or exercise or effect or effectiveness or efficacy)	127
S17	(((cluster N2 headache#) or histamine cephalgia# or horton* syndrome or ciliary neuralgia#)) AND (drug# or pharma* or treat* or therap* or management or intervention# or reduce or reduction or counselling or exercise or effect or effectiveness or efficacy)	832
S16	(trigeminal autonomic N1 cephal*) AND (drug# or pharma* or treat* or therap* or management or intervention# or reduce or reduction or counselling or exercise or effect or effectiveness or efficacy)	118
S15	((tension* N1 headache#) or (idiopathic N1 headache#) or (psychogenic N1 headache#) or (stress N1 headache#)) AND (drug# or pharma* or treat* or therap* or management or intervention# or reduce or reduction or counselling or exercise or effect or effectiveness or efficacy)	1,598
S14	(migraine? or hemicrania) AND (drug# or pharma* or treat* or therap* or management or intervention# or reduce or reduction or counselling or exercise or effect or effectiveness or efficacy)	901
S13	(DE "Migraine Headache") AND (drug# or pharma* or treat* or therap* or management or intervention# or reduce or reduction or counselling or exercise or effect or effectiveness or efficacy)	5,736
S12	((chronic N3 orofacial pain) or (chronic N3 mandibular pain) or (chronic N3 maxillary pain) or (chronic N3 jaw pain) or (chronic N3 face pain) or (chronic N3 facial neuralgia) or	130

	(chronic N3 facial pain) or facialgia) AND (drug# or pharma* or treat* or therap* or management or intervention# or reduce or reduction or counselling or exercise or effect or effectiveness or efficacy)	
S11	(chronic N2 primary headache) AND (drug# or pharma* or treat* or therap* or management or intervention# or reduce or reduction or counselling or exercise or effect or effectiveness or efficacy)	40
S10	((neuralgic shoulder N1 amyotophy) or (post traumatic N1 dystrophy) or (posttraumatic N1 dystrophy) or (reflex sympathetic N1 dystrophy) or autonomic neuropathy or sympathetic dystrophy or (posttraumatic N1 osteoporosis) or (posttraumatic N1 osteopenia)) AND (drug# or pharma* or treat* or therap* or management or intervention# or reduce or reduction or counselling or exercise or effect or effectiveness or efficacy)	348
S9	(algesidystrophy or algodystrophia or algoneurodystrophy or (sudek* N2 atroph*) or (sudek* N2 dystrophy) or (sudek* N2 disease) or (sudek* N2 syndrome)) or (cervical sympathetic N2 dystroph*) or rsd? or (reflex sympathetic N2 dystroph*) or ((shoulder arm N1 syndrome) or (shoulder hand N1 syndrome)) AND (drug# or pharma* or treat* or therap* or management or intervention# or reduce or reduction or counselling or exercise or effect or effectiveness or efficacy)	241
S8	(DE "Neuralgia" OR DE "Trigeminal Neuralgia") AND (drug# or pharma* or treat* or therap* or management or intervention# or reduce or reduction or counselling or exercise or effect or effectiveness or efficacy)	2,232
S7	(((causalgia or (deafferentation N1 pain))) AND (drug# or pharma* or treat* or therap* or management or intervention# or reduce or reduction or counselling or exercise or effect or effectiveness or efficacy)	102
S6	(DE "Complex Regional Pain Syndrome (Type I)") AND (drug# or pharma* or treat* or therap* or management or intervention# or reduce or reduction or counselling or exercise or effect or effectiveness or efficacy)	99
S5	(CRPS or (regional complex N3 pain) or (complex regional N3 pain)) AND (drug# or pharma* or treat* or therap* or management or intervention# or reduce or reduction or counselling or exercise or effect or effectiveness or efficacy)	1,633
S4	(fibromyalgia# or fibrositis or fibrositic nodule) AND (drug# or pharma* or treat* or therap* or management or intervention# or reduce or reduction or counselling or exercise or effect or effectiveness or efficacy)	2,317
S3	(DE "Fibromyalgia") AND (drug# or pharma* or treat* or therap* or management or intervention# or reduce or reduction or counselling or exercise or effect or effectiveness or efficacy)	1,574
S2	(chronic N4 pain#) AND (drug# or pharma* or treat* or therap* or management or intervention# or reduce or reduction or counselling or exercise or effect or effectiveness or efficacy)	16,629

S1	(DE "Chronic Pain") AND (drug# or pharma* or treat* or therap* or management or intervention# or reduce or reduction or counselling or exercise or effect or effectiveness or efficacy)	10,319

Web of Science

Clinicaltrials.gov

1. Condition=chronic pain AND Study type=Interventional (1,740 results)
2. Condition= Fibromyalgia AND Study type=Interventional (604 results)
3. Condition= Chronic widespread pain AND Study type=Interventional (17 results)
4. Condition= Complex regional pain syndrome AND Study type=Interventional (82 results)
5. Condition= "Chronic primary headache" AND Study type=Interventional (1 result)
6. Condition= chronic orofacial pain AND Study type=Interventional (4 results)
7. Condition= chronic migraine AND Study type=Interventional (90 results)
8. Condition= Chronic Tension-Type Headache AND Study type=Interventional (12 results)
9. Condition= Trigeminal autonomic cephalalgias AND Study type=Interventional (46 results)
10. Condition= Chronic temporomandibular disorder pains AND Study type=Interventional (11 results)
11. Condition= Chronic burning mouth AND Study type=Interventional (6 results)
12. Condition= "Chronic primary visceral pain" AND Study type=Interventional (0 results)
13. Condition= "primary chest pain" AND Study type=Interventional (0 results)
14. Condition= "functional chest pain" AND Study type=Interventional (3 results)
15. Condition= Irritable bowel syndrome AND Study type=Interventional (170 results)
16. Condition= Chronic primary abdominal pain syndrome AND Study type=Interventional (1 result)
17. Condition= Bladder pain syndrome AND Study type=Interventional (51 results)
18. Condition= Chronic pelvic pain AND Study type=Interventional (81 results)
19. Condition= Chronic primary musculoskeletal pain AND Study type=Interventional (5 results)
20. Condition= Chronic primary low back pain AND Study type=Interventional (15 results)
21. Condition= Chronic primary cervical pain AND Study type=Interventional (3 results)
22. Condition= Chronic primary thoracic pain AND Study type=Interventional (2 results)
23. Condition= Chronic primary limb pain AND Study type=Interventional (0 results)

OpenTrials

1. Condition=chronic pain NOT Clinicaltrials.gov (1,426 results)
2. Condition= Fibromyalgia NOT Clinicaltrials.gov (524 results)
3. Condition= Chronic widespread pain NOT Clinicaltrials.gov (14 results)
4. Condition= Complex regional pain syndrome NOT Clinicaltrials.gov (97 results)
5. Condition= "Chronic primary headache" NOT Clinicaltrials.gov (2 results)

6. Condition= chronic orofacial pain NOT Clinicaltrials.gov (2 results)
7. Condition= chronic migraine NOT Clinicaltrials.gov (80 results)
8. Condition= Chronic Tension-Type Headache NOT Clinicaltrials.gov (12 results)
9. Condition= Trigeminal autonomic cephalalgias NOT Clinicaltrials.gov (0 results)
10. Condition= Chronic temporomandibular disorder pains NOT Clinicaltrials.gov (2 results)
11. Condition= Chronic burning mouth NOT Clinicaltrials.gov (0 results)
12. Condition= "Chronic primary visceral pain" NOT Clinicaltrials.gov (0 results)
13. Condition= "primary chest pain" NOT Clinicaltrials.gov (0 results)
14. Condition= "functional chest pain" NOT Clinicaltrials.gov (1 results)
15. Condition= Irritable bowel syndrome NOT Clinicaltrials.gov (592 results)
16. Condition= Chronic primary abdominal pain syndrome NOT Clinicaltrials.gov (0 results)
17. Condition= Bladder pain syndrome NOT Clinicaltrials.gov (26 results)
18. Condition= Chronic pelvic pain NOT Clinicaltrials.gov (72 results)
19. Condition= Chronic primary musculoskeletal pain NOT Clinicaltrials.gov (0 results)
20. Condition= Chronic primary low back pain NOT Clinicaltrials.gov (0 results)
21. Condition= Chronic primary cervical pain NOT Clinicaltrials.gov (0 results)
22. Condition= Chronic primary thoracic pain NOT Clinicaltrials.gov (0 results)
23. Condition= Chronic primary limb pain NOT Clinicaltrials.gov (0 results)
